# The association of female reproductive factors with risk of metabolic syndrome in women from NHANES 1999–2018

**DOI:** 10.1186/s12889-023-17207-0

**Published:** 2023-11-21

**Authors:** Ronghua Zuo, Yiting Ge, Jingbo Xu, Lin He, Tao Liu, Bing Wang, Lifang Sun, Shasha Wang, Zhijian Zhu, Yuefei Wang

**Affiliations:** 1 Department of Anesthesiology, Peking University Third Hospital, No. 49, North Garden Street, Haidian District, Beijing, 100191 China; 2 Department of Gynecology, Kunshan Hospital of Traditional Chinese Medicine, No.388 Zuchongzhi Road, Kunshan, Jiangsu 215300 China; 3 Department of Obstetrics and Gynecology, Wuxi No.2 People’s Hospital, 585 Xingyuan North Road, Liangxi District, Wuxi, Jiangsu 214000 China; 4 Department of Cardiology, Jinshan Branch of Shanghai Sixth People’s Hospital, Shanghai, 201500 China

**Keywords:** Metabolic syndrome, Age, Birth, Reproductive factor, Pregnancy

## Abstract

**Background:**

Female reproductive factors such as age at first birth (AFB), age at last birth (ALB), number of pregnancies and live births play an essential role in women’s health. However, few epidemiological studies have evaluated the association between female reproductive factors and metabolic syndrome (MetS). We therefore conducted a cross-sectional study to investigate the association between MetS risk and female reproductive factors.

**Methods:**

We investigated the relationship between AFB, ALB, number of pregnancies and live births and the incidence of MetS using publicly available data from the National Health and Nutrition Examination Survey (NHANES) from 1999 to 2018. Weighted multivariable logistic regression analysis, restricted cubic spline (RCS) model, and subgroup analysis were used to evaluate the association between AFB and ALB and the risk of MetS in women. In addition, the relationship between the number of pregnancies, live births and MetS risk was also explored.

**Results:**

A total of 15,404 women were included in the study, and 5,983 (38.8%) had MetS. RCS models showed an N-shaped relationship between AFB and MetS risk, whereas ALB, number of pregnancies, and live births were linearly associated with MetS. Weighted multivariable logistic regression analysis showed that the number of live births was associated with MetS risk, with ORs of 1.18 (95% CI: 1.04, 1.35) for women with ≥ 5 deliveries compared to women with ≤ 2 births.

**Conclusions:**

AFB was associated with the risk of MetS in an N-shaped curve in women. In addition, women with high live births have a higher incidence of MetS.

## Introduction

Metabolic syndrome (MetS) is a group of clinical syndromes, including abdominal obesity, hyperglycemia, dyslipidemia, and hypertension [1]. The prevalence of MetS in adults is about 37.3% [2], which has become a global disease that seriously affects human health. Studies have found that the prevalence of MetS in China is 25%, with a prevalence of 19.2% in men and 27.0% in women [3]. The risk of cardiovascular disease in patients with MetS is two times higher than that in non-MetS patients, and the risk of death is 1.5 times higher than that in non-MetS patients [4]. Studies have shown that the core pathophysiological basis of MetS is insulin resistance, and many factors, including genes, metabolism, and environmental factors, are related to the occurrence of MetS [5]. Female reproductive factors such as age at first pregnancy (AFB), age at last birth (ALB), number of pregnancies, and live births play an essential role in female health. Previous studies have shown that early AFB is associated with metabolic diseases such as Non-alcoholic fatty liver disease, obesity, and diabetes [6–8]. Epidemiological studies on the relationship between AFB, ALB, number of pregnancies and live births, and MetS are limited, and the results are inconsistent. Lee et al. found that postmenopausal women with more births had a higher risk of developing MetS [9]. However, Moosazadeh et al.’s study showed no relationship between number of pregnancies and MetS [10]. Some studies have shown an increased risk of MetS in postmenopausal women who give birth to their first child early [11, 12], while others have found no association between early AFB and MetS risk [10]. There are few studies on the relationship between female reproductive factors and MetS, and the few available studies have conflicting results; therefore, further validation in a larger population is needed. Thus, the present study aims to investigate the association between female reproductive factors and the risk of MetS using the National Health and Nutrition Examination Survey (NHANES) data from 1999 to 2018 in the United States (U.S.).

## Material and methods

### Study population

The NHANES database is a health and nutrition survey data set for the American population and is a project of the Centers for Disease Control and Prevention [13]. The program aims to provide health and dietary guidance to U.S. residents and improve public health policy. More details about NHANES can be accessed at the website: https://www.cdc.gov/nchs/nhanes/. The NCHS Ethics Review Board approved the protocol and obtained written informed consent from all participants. The subjects of this study were women participants in the NHANES database from 1999 to 2018. Exclusion criteria were: (1) lack of data on AFB, ALB, number of pregnancies, and live births; (2) missing MetS data. A total of 49,209 women participants were included, with missing data on female reproductive factors (*n* = 33,283) and MetS (*n* = 522) excluded, and 15,404 women were ultimately included in the study.

### Reproductive factors

The ages of first and last pregnancies were determined by answering the following questions: “How old were you at the time of your first live birth?” and “How old were you at the time of your last live birth?”. The number of pregnancies was assessed by calculating the number of all pregnancies in the participants, including current pregnancies, live births, miscarriages, stillbirths, tubal pregnancies, or abortions). In contrast, the number of live births was assessed by calculating the total number of pregnancies that resulted in a live birth rather than the number of babies born alive. To evaluate contraceptive use, participants were asked, “Have you ever taken birth control pills for any reason?”. And for using female hormones, participants were asked, “Have you ever used female hormones such as estrogen and progesterone? Please include any form of female hormones, such as pills, creams, patches, and injections, but do not include birth control or use for infertility”. In addition, reproductive factors, including menopausal status, age at menarche, age at menopause, history of hysterectomy, and history of bilateral oophorectomy, were also obtained from the reproductive health questionnaire. Fertility life is the difference between the age of menopause and the age of menarche. We defined pregnancy loss as the difference between the total number of self-reported pregnancies and live births [14]. Further information about this questionnaire data can be found at https://wwwn.cdc.gov/Nchs/Nhanes/2013-2014/RHQ_H.htm#RHQ160.

### MetS ascertainment

MetS can be diagnosed when three of the following five conditions are present, according to criteria proposed by the American Endocrine Society and the American Society of Clinical Endocrinology [15]: (1) waist circumference (WC) elevation (≥ 88 cm in women and ≥ 102 cm in men), (2) elevated triglycerides (TG, ≥ 150 mg/dL) or drug-treated TG, (3) low high-density lipoprotein-cholesterol (HDL-C, < 40 mg/dL for men and < 50 mg/dL for women) or use of drugs for low HDL-C, (4) elevated blood pressure (systolic blood pressure (SBP) ≥ 130 mmHg or diastolic blood pressure (DBP) ≥ 85 mmHg or both) or antihypertensive drug use, (5) elevated fast glucose ≥ 100 mg/dL or drug therapy for hyperglycemia.

### Covariates

The study incorporated a range of covariates for analysis, including demographic variables (age, race, family income-to-poverty ratio (PIR), education level, and marital status), questionnaire data (diabetes mellitus (DM), smoker, coronary heart disease (CHD), alcohol user, angina pectoris, heart attack, congestive heart failure (CHF), hypertension and stroke), dietary data (mean energy intake), and laboratory data (hemoglobin (Hb), fast blood glucose (FBG), serum creatinine (Scr), glycosylated hemoglobin (HbA1c), uric acid (UA), total cholesterol (TC), triglycerides (TG), blood urea nitrogen (BUN), HDL-C, estimated glomerular filtration rate (eGFR)), Examination data (body mass index (BMI), WC, and blood pressure). Individuals who smoked less than 100 cigarettes during their lifetime, those who smoked more than 100 cigarettes in their lifetime but were not currently smokers, and those who smoked more than 100 cigarettes in their lifetime or who now smoked every day or several days were defined as nonsmokers, former and current smokers, respectively. Detailed covariates information is publicly obtained from the NHANES database (https://www.cdc.gov/nchs/nhanes/).

### Statistical analysis

All NHANES estimations were sample weights computed. According to data distribution characteristics, continuous variables use mean ± standard deviation or interquartile range to describe the trend of data concentration, and categorical variables use frequency to describe. Weighted T-tests or Mann-Whitney U test were used to compare between-group differences for continuous variables. Weighted multivariable logistic regression analysis was used to explore the association between AFB, ALB, number of pregnancies, live births and MetS. Model 1 adjusted for age and race/ethnicity, and Model 2 adjusted for age, race/ethnicity, education level, marriage status, family PIR, hypertension, smoking, and alcohol use. Model 3 was based on Model 2 with adjustments for CHD, CHF, heart attack, angina pectoris and stroke, age at menopause, BMI, WC, SBP, DBP, mean energy intake, Hb, FBG, HbA1c, menopausal status, age at menarche, oral contraceptives, use of female hormones, previous hysterectomy, bilateral ovariectomy, BUN, UA, Scr, eGFR, TC, TG, HDL-C, number of live births and pregnancies, pregnancy loss and fertile lifespan. Based on Model 3, subgroup analyses were performed to examine whether the effects of AFB and ALB on MetS could be changed by age, race, menopausal status, hysterectomy, female hormone use, age at menarche, or reproductive age. In addition, restrictive cubic splines (RCS) were used to analyze the association between AFB, ALB, number of live births, pregnancies and MetS. Statistical analysis was performed using Rstudio 3.6.4 and SPSS 22.0. *P-*value < 0.05 was considered statistically significant.

## Results

### Baseline characteristics

The overall prevalence of MetS in the included study population was 38.8% (5983/15404). The average age of MetS patients was significantly higher than that of non-MetS population (*P* < 0.001).

The mean values of WC, SBP, DBP, FBG, TG, and HDL-C in patients with MetS were 107.19 ± 0.27 cm, 129.50 ± 0.40 mmHg, 71.83 ± 0.30 mmHg, 119.84 ± 0.78 mg/dL, 183.38 ± 2.30 mg/dL and 46.25 ± 0.22 mg/dL, respectively. There were significant differences in AFB, number of live births, and number of pregnancies between non-MetS and MetS participants (*P* < 0.001). The baseline characteristics of the study population are shown in Table 1.

**Table 1:** Demographic characteristics of the study participants

Variables	Overall (*n* = 15,404)	Non-MetS (*n* = 9,421)	MetS (*n* = 5,983)	*P*-value
Age, years	52.77 ± 0.19	50.22 ± 0.23	57.49 ± 0.26	< 0.001
Race, n (%)				0.067
Mexican American	2950 (19.2%)	1677 (10.9%)	1273 (8.3%)	
Other Hispanic	1386 (9.0%)	786 (5.1%)	600 (3.9%)	
Non-Hispanic Black	3151 (20.5%)	2021 (13.1%)	1130 (7.3%)	
Non-Hispanic White	6757 (43.9%)	4165 (27.0%)	2592 (16.8%)	
Other race	1160 (7.5%)	772 (5.0%)	388 (2.5%)	
Family PIR	2.82 ± 0.03	2.96 ± 0.03	2.56 ± 0.04	< 0.001
Education level, n (%)				< 0.001
High school	4615 (30.0%)	2506 (16.3%)	2109 (13.7%)	
College	1521 (9.9%)	851 (5.5%)	670 (4.4%)	
Graduate	9268 (60.2%)	6064 (39.4%)	3204 (20.8%)	
Marital status, n (%)				< 0.001
Having a partner	9046 (58.7%)	5777 (37.5%)	3269 (21.2%)	
No partner	5279 (34.3%)	2888 (18.7%)	2391 (15.6%)	
Unmarried	1079 (7.0%)	756 (4.9%)	323 (2.1%)	
Hypertension, n (%)				< 0.001
No	7809 (50.7%)	6077 (39.5%)	1732 (11.2%)	
Yes	7595 (49.3%)	3344 (21.7%)	4251 (27.6%)	
DM, n (%)				< 0.001
No	12,380 (80.4%)	8770 (56.9%)	3610 (23.4%)	
Yes	3024 (19.6%)	651 (4.2%)	2373 (15.4%)	
Smoker, n (%)				< 0.001
No	9567 (62.1%)	5982 (38.8%)	3585 (23.3%)	
Former	3124 (20.3%)	1771 (11.5%)	1353 (8.8%)	
Now	2713 (17.6%)	1668 (10.8%)	1045 (6.8%)	
Alcohol user, n (%)				< 0.001
No	3456 (22.4%)	1909 (12.4%)	1547 (10.0%)	
Former	2894 (18.8%)	1506 (9.8%)	1388 (9.0%)	
Mild	4427 (28.7%)	2861 (18.6%)	1566 (10.1%)	
Moderate	2609 (16.9%)	1773 (11.5%)	836 (5.4%)	
Heavy	2018 (13.1%)	1372 (8.9%)	646 (4.2%)	
CHD, n (%)				< 0.001
No	14,868 (96.5%)	9240 (60.0%)	5628 (36.5%)	
Yes	536 (3.5%)	181 (1.2%)	355 (2.3%)	
CHF, n (%)				< 0.001
No	14,867 (96.5%)	9231 (59.9%)	5636 (36.6%)	
Yes	537 (3.5%)	190 (1.2%)	347 (2.3%)	
Angina pectoris, n (%)				< 0.001
No	14,907 (96.8%)	9239 (60.0%)	5668 (36.8%)	
Yes	497 (3.2%)	182 (1.2%)	315 (2.0%)	
Heart attack, n (%)				< 0.001
No	14,814 (96.2%)	9197 (59.7%)	5617 (36.5%)	
Yes	590 (3.8%)	224 (1.5%)	366 (2.4%)	
Stroke, n (%)				< 0.001
No	14,702 (95.4%)	9120 (59.2%)	5582 (36.2%)	
Yes	702 (4.6%)	301 (2.0%)	401 (2.6%)	
Menopause status, n (%)				< 0.001
No	2584 (16.8%)	2004 (13.0%)	580 (3.8%)	
Yes	12,820 (83.2%)	7417 (48.1%)	5403 (35.1%)	
Oral contraceptive use, n (%)				< 0.001
No	5349 (34.7%)	3138 (20.4%)	2211 (14.4%)	
Yes	10,055 (65.3%)	6283 (40.8%)	3772 (24.5%)	
Use female hormones, n (%)				< 0.001
No	11,635 (75.5%)	7350 (47.7%)	4285 (27.8%)	
Yes	3769 (24.5%)	2071 (13.4%)	1698 (11.0%)	
Had a hysterectomy, n (%)				< 0.001
No	10,886 (70.7%)	7154 (46.4%)	3732 (24.2%)	
Yes	4518 (29.3%)	2267 (14.7%)	2251 (14.6%)	
Both ovaries removed, n (%)				< 0.001
No	12,658 (82.2%)	8048 (52.2%)	4610 (29.9%)	
Yes	2746 (17.8%)	1373 (8.9%)	1373 (8.9%)	
BMI, kg/m^2^	29.31 ± 0.09	27.38 ± 0.10	32.89 ± 0.13	< 0.001
Waist circumference, cm	97.29 ± 0.22	92.10 ± 0.23	106.89 ± 0.26	< 0.001
SBP, mmHg	123.60 ± 0.24	120.43 ± 0.28	129.49 ± 0.35	< 0.001
DBP, mmHg	70.30 ± 0.18	69.87 ± 0.17	71.09 ± 0.27	< 0.001
Hb, g/dL	13.49 ± 0.02	13.44 ± 0.02	13.59 ± 0.03	< 0.001
Mean energy	1751.33 ± 6.98	1775.89 ± 8.79	1705.80 ± 11.03	< 0.001
Intake (kcal/day)				< 0.001
FBG, mg/dL	104.90 ± 0.31	96.98 ± 0.21	119.57 ± 0.66	< 0.001
HbA1c, %	5.64 ± 0.01	5.41 ± 0.01	6.07 ± 0.02	< 0.001
TC, mg/dL	201.68 ± 0.52	199.60 ± 0.57	205.53 ± 0.82	< 0.001
TG, mg/dL	123.29 ± 0.99	97.02 ± 0.67	171.98 ± 2.23	< 0.001
HDL-C, mg/dL	58.14 ± 0.24	63.43 ± 0.28	48.31 ± 0.25	< 0.001
BUN, mg/dL	13.44 ± 0.08	12.81 ± 0.09	14.62 ± 0.12	< 0.001
Scr, mg/dL	0.79 ± 0.00	0.76 ± 0.00	0.83 ± 0.01	< 0.001
UA, mg/dL	4.85 ± 0.01	4.54 ± 0.01	5.44 ± 0.02	< 0.001
eGFR, ml/min/1.73m^2^	90.27 ± 0.32	93.42 ± 0.36	84.44 ± 0.43	< 0.001
AFB, years	22.60 ± 0.09	22.98 ± 0.12	21.89 ± 0.09	< 0.001
ALB, years	29.22 ± 0.08	29.22 ± 0.10	29.22 ± 0.10	0.970
Number of pregnancies, times	3.60 ± 0.02	3.48 ± 0.02	3.81 ± 0.04	< 0.001
Number of live births, times	2.87 ± 0.02	2.76 ± 0.02	3.08 ± 0.03	< 0.001
Pregnancy loss, times	0.73 ± 0.02	0.72 ± 0.02	0.73 ± 0.03	0.818
Age at menarche, years	12.74 ± 0.02	12.79 ± 0.02	12.66 ± 0.03	< 0.001
Age at menopause, years	41.97 ± 0.13	41.44 ± 0.17	42.97 ± 0.15	< 0.001
Fertile lifespan, years	29.23 ± 0.14	28.65 ± 0.17	30.30 ± 0.15	< 0.001

Data are presented as mean ± SD or n (%)

*Abbreviations*: *MetS* Metabolic syndrome, *DM* Diabetes mellitus, *BMI* Body mass index, *CHD* Coronary heart disease, *CHF* Congestive heart failure, *SBP* Systolic blood pressure, *DBP* Diastolic blood pressure, *Hb* Hemoglobin, *FBG* Fast glucose, *HbA1c* Glycosylated hemoglobin, *TC* Total cholesterol, *TG* Triglycerides, *HDL-cholesterol* High density lipoprotein-cholesterol, *BUN* Blood urea nitrogen, *UA* Uric acid, *Scr* Serum creatinine, *eGFR* Estimated glomerular filtration rate, *AFB* Age at first birth, *ALB* Age at last birth

### Association between AFB, ALB, number of pregnancies, live births, and MetS

**Fig. 1:**
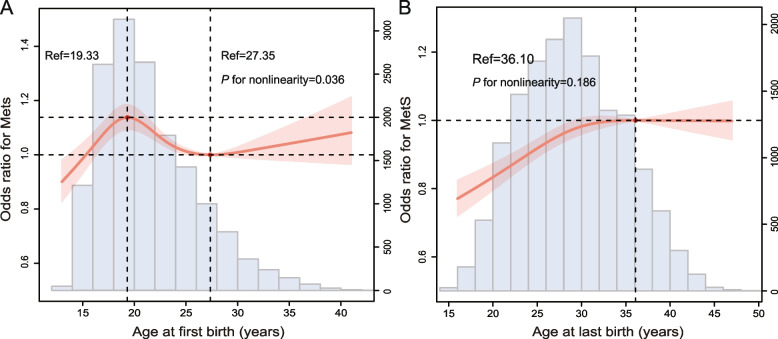
Restricted cubic spline plots of associations between **A** AFB, **B** ALB and prevalence of MetS. Abbreviation: AFB, age at first birth; ALB, age at last birth; MetS, metabolic syndrome

**Fig. 2:**
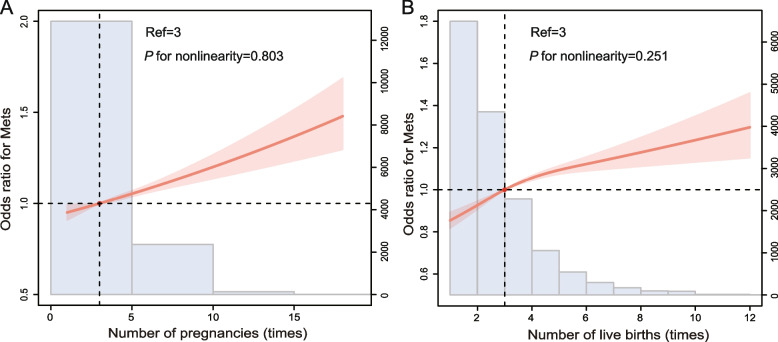
Restricted cubic spline plots of associations between **A** number of pregnancies and **B** live births and prevalence of MetS. Abbreviation: MetS, metabolic syndrome

We used the RCS fitted model to plot the change in risk of MetS with increasing AFB, ALB, number of pregnancies and live births. After adjusting for covariates, the association between AFB and MetS showed a nonlinear relationship, peaking at about 19 years of age and the risk of MetS decreasing as the AFB increased with an N-shaped curve (*P* for nonlinearity = 0.036, Fig. 1A). However, the association between ALB, number of pregnancies, and live births and MetS was a linear relationship, and the risk of MetS increased with increasing ALB, number of pregnancies and live births (ALB, *P* for nonlinearity = 0.186, Fig. 1B; the number of pregnancies, *P* for nonlinearity = 0.803, Fig. 2A; the number of live births, *P* for nonlinearity = 0.251, Fig. 2B). The relationship between AFB, ALB, and the risk of MetS is presented in Table 2. In studying the association between AFB and MetS, patients were divided into eight groups based on age: < 18, 18–20, 21–23, 24–26, 27–29, 30–32, 33–35, and ≥ 36 years. Weighted multivariable logistic regression analysis showed that after adjusting for race and age, participants with AFB of 21–23, 24–26, 27–29, 30–32, 33–35, and ≥ 36 had a significantly lower risk of MetS compared with those with AFB < 18, with ORs of 0.81 (95% CI: 0.72, 0.90), 0.71 (95% CI: 0.63, 0.80), 0.67 (95% CI: 0.58, 0.77), 0.58 (95% CI: 0.47, 0.70), 0.59 (95% CI: 0.47, 0.70) and 0.50 (95% CI: 0.35, 0.71), respectively (*P* for trend < 0.001). After further adjustment for covariates in models 2 and 3, AFB was not associated with an increased risk of MetS. In addition, in studying the association between ALB and MetS, participants were grouped into ≤ 24, 25–29, 30–34, 35–39, and ≥ 40 years, and ALB was not associated with the risk of MetS in model 2 and 3 (Table 2). Second, we divided the participants into four groups according to the number of pregnancies and live births: ≤ 2, 3, 4, and ≥ 5. The risk of MetS was significantly higher in participants with more than four pregnancies in model 1, but statistical significance was not maintained after adjustment for covariates in models 2 and 3. Notably, the greater the number of live births, the higher the risk of MetS in model 1. After further adjustment for confounding factors, the risk of having MetS was significantly higher in the participants whose number of live births was ≥ 5 in models 2 and 3 (Table 3).

**Table 2:** Associations of AFB, and ALB with the risk of MetS

	Model 1		Model 2		Model 3	
	OR (95%CI)	*P* for trend	OR (95%CI)	*P* for trend	OR (95%CI)	*P* for trend
AFB		< 0.001		0.009		0.791
< 18	1.00		1.00		1.00	
18–20	0.97 (0.87, 1.07)		1.08 (0.96, 1.22)		1.08 (0.95, 1.23)	
21–23	0.81 (0.72, 0.90)***		0.96 (0.85, 1.09)		1.03 (0.89, 1.18)	
24–26	0.71 (0.63, 0.80)***		0.92 (0.80, 1.07)		0.99 (0.85, 1.16)	
27–29	0.67 (0.58, 0.77)***		0.94 (0.80, 1.11)		1.10 (0.91, 1.32)	
30–32	0.58 (0.47, 0.70)***		0.92 (0.74, 1.15)		1.11 (0.87, 1.40)	
33–35	0.59 (0.47, 0.70)***		0.88 (0.66, 1.17)		0.98 (0.71, 1.35)	
≥ 36	0.50 (0.35, 0.71)***		0.74 (0.50, 1.09)		0.81 (0.53, 1.25)	
ALB		< 0.001		0.005		0.402
≤ 24	1.00		1.00		1.00	
25–29	0.95 (0.87, 1.05)		1.07 (0.97, 1.20)		1.09 (0.97, 1.22)	
30–34	0.87 (0.79, 0.96)*		1.05 (0.94, 1.17)		1.10 (0.97, 1.25)	
35–39	0.88 (0.79, 0.98)*		1.02 (0.90, 1.16)		1.08 (0.94, 1.25)	
≥ 40	0.85 (0.72, 1.01)		0.93 (0.77, 1.13)		1.08 (0.88, 1.33)	

Model 1: age and race/ethnicity

Model 2: model 1 variables plus education level, marriage status, family PIR, hypertension, smoking, and alcohol use

Model 3 was adjusted for model 2 variables plus coronary heart disease, congestive heart-failure, heart attack, angina and stroke, age at menopause, body mass index, waist circumference, systolic blood pressure, diastolic blood pressure, mean energy intake, hemoglobin, fast glucose, glycosylated hemoglobin, menopausal status, age at menarche, oral contraceptives, use of female hormones, previous hysterectomy, bilateral ovariectomy, blood urea nitrogen, uric acid, serum creatinine, estimated glomerular filtration rate, total cholesterol, triglyceride, high-density lipoprotein-cholesterol, number of live births and pregnancies, pregnancy loss and fertile lifespan

*Abbreviations*: *AFB* Age at first birth, *ALB* Age at last birth, *MetS* Metabolic syndrome, *OR* Odds ratio, *CI* Confidence interval

^*^P < 0.05, **P < 0.01, ***P < 0.001

**Table 3:** Associations of number of pregnancies, and number of live births with the risk of MetS

	Model 1		Model 2		Model 3	
	OR (95%CI)	*P* for trend	OR (95%CI)	*P* for trend	OR (95%CI)	*P* for trend
Number of pregnancies		< 0.001		0.032		0.145
≤ 2	1.00		1.00		1.00	
3	1.01 (0.91, 1.09)		1.01 (0.89, 1.09)		1.01 (0.86, 1.08)	
4	1.12 (1.02, 1.24)*		1.08 (0.97, 1.21)		1.04 (0.92, 1.17)	
≥ 5	1.22 (1.12, 1.34)***		1.10 (0.99, 1.22)		1.08 (0.96, 1.22)	
Number of live births		< 0.001		0.002		0.021
≤ 2	1.00		1.00		1.00	
3	1.08 (0.99, 1.17)		1.05 (0.95, 1.15)		1.04 (0.94, 1.15)	
4	1.21 (1.10, 1.34)***		1.09 (0.97, 1.22)		1.05 (0.93, 1.19)	
≥ 5	1.40 (1.26, 1.56)***		1.21 (1.07, 1.37)**		1.18 (1.04, 1.35)**	

Model 1: age and race/ethnicity

Model 2: model 1 variables plus education level, marriage status, family PIR, hypertension, smoking, and alcohol use

Model 3 was adjusted for model 2 variables plus coronary heart disease, congestive heart-failure, heart attack, angina and stroke, age at menopause, body mass index, waist circumference, systolic blood pressure, diastolic blood pressure, mean energy intake, hemoglobin, fast glucose, glycosylated hemoglobin, menopausal status, age at menarche, oral contraceptives, use of female hormones, previous hysterectomy, bilateral ovariectomy, blood urea nitrogen, uric acid, serum creatinine, estimated glomerular filtration rate, total cholesterol, triglyceride, high-density lipoprotein-cholesterol, number of live births and pregnancies, pregnancy loss and fertile lifespan

*Abbreviations*: *MetS* Metabolic syndrome, *OR* Odds ratio, *CI* Confidence interval

^*^P < 0.05, **P < 0.01, ***P < 0.001

### Subgroup analyses

Table 4 shows a stronger association between AFB and MetS among participants younger than 45 years, Mexican Americans and other ethnicities, and women who had hysterectomy. Additionally, there was a significant interaction for most subgroups (*P* for interaction < 0.05). And a stronger association between ALB and MetS was found in other Hispanic individuals, older at menarche, non-hysterectomized, and with a fertile lifespan > 35 years, respectively (Table 5). Notably, the association differed among the subgroups for age, race, menopausal status, hysterectomy, age at menarche, female hormone use and fertile lifespan (*P* for interaction < 0.05).

**Table 4:** Subgroups analysis for the associations of AFB with the risk of MetS

	< 18	18–20	21–23	24–26	27–29	30–32	33–35	≥ 36	*P* for trend	*P* for interaction
	OR (95%CI)	OR (95%CI)	OR (95%CI)	OR (95%CI)	OR (95%CI)	OR (95%CI)	OR (95%CI)	OR (95%CI)		
Age										0.006
45	1.00	1.02 (0.81, 1.28)	0.99 (0.76, 1.28)	0.83 (0.60, 1.14)	1.10 (0.77, 1.57)	1.09 (0.68, 1.76)	2.01 (1.11, 3.65)*	0.46 (0.14, 1.48)	0.641	
≥ 45	1.00	1.10 (0.94, 1.29)	1.03 (0.87, 1.21)	1.03 (0.86, 1.24)	1.07 (0.86, 1.24)	1.08 (0.82, 1.43)	0.76 (0.53, 1.11)	0.87 (0.55, 1.40)	0.373	
Race										< 0.001
Mexican	1.00	1.16 (0.90, 1.50)	1.32 (1.00, 1.74)*	1.25 (0.90, 1.74)	1.22 (0.81, 1.83)	0.98 (0.53, 1.78)	1.00 (0.44, 2.27)	1.36 (0.51, 3.65)	0.391	
American										
Other	1.00	1.08 (0.72, 1.62)	1.09 (0.70, 1.70)	1.16 (0.70, 1.93)	0.99 (0.52, 1.90)	1.66 (0.75, 3.66)	1.06 (0.39, 2.91)	1.42 (0.47, 4.33)	0.422	
Hispanic										
Non-Hispanic	1.00	0.88 (0.68, 1.12)	0.83 (0.62, 1.10)	0.72 (0.49, 1.04)	1.19 (0.75, 1.89)	1.32 (0.72, 2.44)	1.17 (0.54, 2.56)	0.78 (0.23, 2.62)	0.887	
Black										
Non-Hispanic	1.00	1.05 (0.83, 1.34)	0.99 (0.77, 1.27)	0.98 (0.75, 1.29)	1.01 (0.75, 1.29)	0.93 (0.64, 1.37)	0.92 (0.56, 1.50)	0.69 (0.35, 1.39)	0.315	
White										
Other race	1.00	2.04 (1.08, 3.84)*	1.02 (0.53, 1.96)	0.91 (0.47, 1.78)	1.54 (0.77, 3.10)	1.69 (0.78, 3.68)	0.87 (0.30, 2.51)	0.30 (0.05, 1.82)	0.576	
Menopause status										0.001
No	1.00	0.88 (0.63, 1.24)	0.83 (0.57, 1.21)	0.69 (0.45, 1.07)	0.98 (0.61, 1.57)	0.82 (0.44, 1.53)	1.66 (0.80, 3.46)	0.41 (0.13, 1.24)	0.703	
Yes	1.00	1.11 (0.96, 1.28)	1.05 (0.90, 1.22)	1.04 (0.87, 1.23)	1.11 (0.91, 1.36)	1.15 (0.89, 1.48)	0.84 (0.59, 1.19)	0.90 (0.56, 1.45)	0.766	
Hysterectomy										0.001
No	1.00	1.00 (0.85, 1.17)	1.04 (0.87, 1.23)	0.87 (0.72, 1.05)	1.03 (0.83, 1.28)	1.10 (0.84, 1.44)	0.98 (0.68, 1.41)	0.79 (0.49, 1.28)	0.687	
Yes	1.00	1.25 (1.00, 1.56)*	1.01 (0.80, 1.29)	1.31 (0.99, 1.73)	1.30 (0.90, 1.86)	1.07 (0.63, 1.83)	0.90 (0.45, 1.80)	0.79 (0.27, 2.29)	0.753	
Female hormone										0.005
No	1.00	1.10 (0.95, 1.27)	1.03 (0.88, 1.20)	0.91 (0.76, 1.10)	0.99 (0.81, 1.23)	1.04 (0.79, 1.36)	1.05 (0.73, 1.50)	0.84 (0.52, 1.34)	0.313	
Yes	1.00	1.02 (0.77, 1.35)	1.05 (0.78, 1.42)	1.28 (0.92, 1.78)	1.51 (0.92, 1.78)	1.29 (0.78, 2.14)	0.71 (0.34, 1.45)	0.59 (0.21, 1.64)	0.243	
Age at menarche										0.151
12	1.00	1.09 (0.83, 1.42)	1.02 (0.76, 1.37)	1.10 (0.78, 1.55)	1.09 (0.72, 1.65)	1.37 (0.79, 2.35)	0.75 (0.37, 1.54)	0.75 (0.28, 2.03)	0.979	
12–13	1.00	1.15 (0.96, 1.38)	1.04 (0.86, 1.27)	0.99 (0.79, 1.23)	1.08 (0.84, 1.41)	0.98 (0.70, 1.37)	1.02 (0.64, 1.61)	0.74 (0.39, 1.42)	0.371	
13	1.00	0.98 (0.75, 1.27)	1.00 (0.76,1.32)	0.97 (0.71, 1.32)	1.10 (0.78, 1.56)	1.17 (0.75, 1.81)	1.04 (0.58, 1.86)	0.93 (0.45, 1.90)	0.562	
Fertile lifespan										0.001
28	1.00	1.07 (0.88, 1.29)	0.99 (0.79, 1.22)	1.04 (0.80, 1.35)	1.12 (0.81, 1.54)	1.23 (0.79, 1.90)	1.06 (0.55, 2.04)	0.64 (0.21, 1.90)	0.725	
28–35	1.00	1.16 (0.97, 1.39)	1.05 (0.87, 1.28)	1.01 (0.81, 1.26)	1.11 (0.86, 1.44)	1.00 (0.72, 1.41)	1.05 (0.67, 1.66)	0.76 (0.40, 1.47)	0.529	
35	1.00	0.98 (0.75, 1.28)	1.00 (0.76, 1.33)	0.98 (0.72, 1.34)	1.11 (0.78, 1.57)	1.18 (0.76, 1.84)	1.06 (0.60, 1.89)	0.95 (0.46, 1.95)	0.491	

Analyses was adjusted for age, race/ethnicity, education level, marriage status, family PIR, hypertension, smoking, and alcohol use, coronary heart disease, congestive heart-failure, heart attack, angina and stroke, age at menopause, body mass index, waist circumference, systolic blood pressure, diastolic blood pressure, mean energy intake, hemoglobin, fast glucose, glycosylated hemoglobin, menopausal status, age at menarche, oral contraceptives, use of female hormones, previous hysterectomy, bilateral ovariectomy, blood urea nitrogen, uric acid, serum creatinine, estimated glomerular filtration rate, total cholesterol, triglyceride, high-density lipoprotein-cholesterol, number of live births and pregnancies, pregnancy loss and fertile lifespan

All *P*-values were calculated using < 18 as the reference

*Abbreviations*: *AFB* Age at first birth, *MetS* Metabolic syndrome, *OR* Odds ratio, *CI* Confidence interval

^*^P < 0.05

**Table 5:** Subgroups analysis for the associations of ALB with the risk of MetS

	≤ 24	25–29	30–34	35–39	≥ 40	*P* for trend	*P* for interaction
	OR (95%CI)	OR (95%CI)	OR (95%CI)	OR (95%CI)	OR (95%CI)		
Age							0.020
45	1.00	1.22 (0.99, 1.52)	1.22 (0.96, 1.55)	1.15 (0.84, 1.58)	1.24 (0.64, 2.42)	0.925	
≥ 45	1.00	1.00 (0.86, 1.15)	1.01 (0.87, 1.17)	1.01 (0.86, 1.18)	1.00 (0.80, 1.25)	0.320	
Race							< 0.001
Mexican	1.00	1.14 (0.86, 1.51)	1.07 (0.80, 1.43)	1.11 (0.81, 1.52)	1.35 (0.90, 2.04)	0.526	
American							
Other	1.00	1.64 (1.10, 2.44)*	1.22 (0.80, 1.84)	1.77 (1.13, 2.76)*	0.81 (0.37, 1.79)	0.701	
Hispanic							
Non-Hispanic	1.00	0.97 (0.76, 1.25)	0.95 (0.73, 1.25)	1.04 (0.76, 1.42)	1.02 (0.62, 1.68)	0.885	
Black							
Non-Hispanic	1.00	1.10 (0.92, 1.32)	1.17 (0.97, 1.42)	1.05 (0.84, 1.32)	1.12 (0.79, 1.58)	0.106	
White							
Other Race	1.00	0.82 (0.49, 1.37)	1.14 (0.69, 1.87)	0.84 (0.48, 1.48)	0.67 (0.30, 1.47)	0.559	
Menopause status							0.014
No	1.00	1.22 (0.88, 1.71)	1.10 (0.77, 1.58)	1.23 (0.79, 1.90)	0.91 (0.45, 1.86)	0.576	
Yes	1.00	1.04 (0.92, 1.18)	1.03 (0.93, 1.22)	1.03 (0.88, 1.19)	1.05 (0.84, 1.31)	0.565	
Hysterectomy							0.039
No	1.00	1.15 (0.99, 1.33)	1.20 (1.03, 1.39)*	1.15 (0.97, 1.36)	1.08 (0.84, 1.37)	0.245	
Yes	1.00	1.00 (0.83, 1.22)	0.95 (0.76, 1.17)	0.99 (0.76, 1.29)	1.15 (0.76, 1.75)	0.721	
Female hormone							0.025
No	1.00	1.11 (0.97, 1.28)	1.05 (0.91, 1.21)	1.10 (0.93, 1.29)	1.04 (0.82, 1.31)	0.179	
Yes	1.00	1.03 (0.82, 1.31)	1.27 (0.99, 1.63)	1.02 (0.76, 1.36)	1.20 (0.75, 1.93)	0.430	
Age at Menarche							0.012
12	1.00	1.17 (0.92, 1.50)	1.14 (0.88, 1.49)	0.97 (0.71, 1.34)	1.09 (0.66, 1.80)	0.94	
12–13	1.00	0.99 (0.84, 1.16)	1.05 (0.88, 1.24)	0.95 (0.78, 1.16)	0.97 (0.72, 1.31)	0.341	
13	1.00	1.26 (0.99, 1.60)	1.21 (0.95, 1.55)	1.41 (1.08, 1.83)*	1.35 (0.93, 1.96)	0.762	
Fertile lifespan							0.006
28	1.00	1.06 (0.89, 1.26)	1.01 (0.83, 1.23)	1.04 (0.80, 1.35)	1.06 (0.61, 1.85)	0.778	
28–35	1.00	1.00 (0.85, 1.18)	1.07 (0.90, 1.27)	0.98 (0.81, 1.20)	1.01 (0.75, 1.36)	0.406	
35	1.00	1.26 (0.99, 1.60)	1.22 (0.96, 1.56)	1.43 (1.10, 1.86)*	1.38 (0.95, 2.00)	0.786	

Analyses was adjusted for age, race/ethnicity, education level, marriage status, family PIR, hypertension, smoking, and alcohol use, coronary heart disease, congestive heart-failure, heart attack, angina and stroke, age at menopause, body mass index, waist circumference, systolic blood pressure, diastolic blood pressure, mean energy intake, hemoglobin, fast glucose, glycosylated hemoglobin, menopausal status, age at menarche, oral contraceptives, use of female hormones, previous hysterectomy, bilateral ovariectomy, blood urea nitrogen, uric acid, serum creatinine, estimated glomerular filtration rate, total cholesterol, triglyceride, high-density lipoprotein-cholesterol, number of live births and pregnancies, pregnancy loss and fertile lifespan

All *P*-values were calculated using ≤ 24 as the reference

*Abbreviations*: *ALB* Age at last birth, *MetS* Metabolic syndrome, *OR* Odds ratio, *CI* Confidence interval

^*^P < 0.05

## Discussion

This study is a cross-sectional study of female participants with information on female reproductive factors and MetS in the NHANES database (1999–2018). We observed N-shaped relationships between MetS risk and AFB. The prevalence of MetS was significantly higher in participants with ≥ 5 deliveries compared to those with ≤ 2 births (OR = 1.18, 95% CI: 1.04, 1.35). Further analysis using RCS models showed a linear relationship between increased ALB, increased number of pregnancies and live births and risk of MetS.

The incidence of MetS in women in this study was similar to that in Zhou et al. [16]. Some evidence also suggests that younger AFB is associated with an increased risk of metabolic disease [17–19]. Sim et al. showed that early AFB was associated with an elevated risk of MetS in postmenopausal women [12]. A possible mechanism is that younger AFB is related to various components of metabolic disorders, such as increased BMI [8], elevated blood pressure and triglyceride [12]. In addition, women with a first child at a younger age are likely to be less educated and have a lower economic level, which may be accompanied by poorer nutritional quality, thereby increasing the incidence of MetS [20]. The results of a large cohort study in Iran showed no association between AFB and the development of MetS [10]. Different findings may be related to different populations included in the study. Therefore, the correlation between AFB and MetS risk needs to be confirmed by further research. Few studies have examined the association between ALB and the prevalence of MetS. Our findings suggest a linear relationship between ALB and MetS risk. However, Shin et al. showed that a younger age at the last birth was associated with an increased risk of MetS in postmenopausal women [21]. Previous studies have shown that the risk of DM was significantly reduced at the later ALB [22]. The relationship between ALB and MetS and their components needs to be further studied. Moosazadeh et al. showed no association between number of pregnancies and MetS, but more pregnancies were a risk factor for increased WC in women [10]. Our results suggest that women with more than 5 live births were 1.18 times more likely to develop MetS than those with 2 live births, whereas there was no similar association for the number of pregnancies. Complete labor has a more significant impact on female hormones than pregnancy. Wu et al. showed that parity was associated with a 52% higher risk of MetS in women who gave birth four or more times [23]. Several studies in China also support a positive correlation between multiple births and MetS [23, 24]. However, shi et al. showed that multiparity was unrelated to MetS in normal-weight postmenopausal women [25]. The possible mechanisms are as follows. (i) Pregnancy-related weight gain can lead to subsequent obesity [26]. Pregnancy may also induce unhealthy behaviors, such as less activity and excessive caloric intake, which may also contribute to obesity. The duration of obesity is a significant risk factor for diabetes [27]. (ii) Pregnancy is characterized by increased adipose tissue and lipolysis, insulin resistance, and inflammation [28, 29], which may persist after delivery. (iii) Moreover, pregnancy-related complications such as gestational diabetes mellitus and gestational hypertension are associated with type 2 diabetes (T2DM) and cardiovascular disease in later life [30, 31]. Multiple births are also considered a risk factor for T2DM in later life [7, 32].

Our study has some strengths. First, this study explored the relationship between four important female reproductive factors and MetS, providing evidence for reducing the incidence of MetS in women. Secondly, this study has a large sample size and many covariates to ensure the reliability of the results. However, this study also has some limitations. We did not analyze the relationship between female reproductive factors and the components of MetS. Data on midwifery characteristics, pre- and post-pregnancy BMI, and breastfeeding history per pregnancy were not available, and these data may impact MetS risk in the future [33]. Then, as this study is a cross-sectional study, the exact mechanism between reproductive factors and MetS is unclear, and more trials are needed to discover the association of female reproductive factors with the incidence of MetS in the future. Finally, our study population was derived from the NHANES database, so the findings may not be appropriate for other races worldwide.

## Conclusion

Our results demonstrated that AFB displayed an N-curve association with MetS, while ALB, number of pregnancies, and live births were positively associated with MetS based on the large cross-sectional study. In order to reduce the risk of MetS in women, further research is needed to focus on the potential mechanisms of the relationship between female reproductive factors and MetS.

## Data Availability

The datasets used and/or analysed during the current study available from the corresponding author on reasonable request.
